# Study on Method for Measuring Coating Emissivity by Applying Active Irradiation Based on Infrared Thermal Imager

**DOI:** 10.3390/s22062392

**Published:** 2022-03-20

**Authors:** Yiwen Li, Puyousen Zhang, Ge Chen, Yao Li, Weizhuo Hua, Yuqin Li, Zhaoqiang Jiao

**Affiliations:** Science and Technology on Plasma Dynamics Laboratory, Air Force Engineering University, Xi’an 710038, China; alpsen@foxmail.com (P.Z.); 18189144368@163.com (G.C.); liyao_0927@163.com (Y.L.); hwz1991@sina.com (W.H.); yuqin511@163.com (Y.L.); jiao_zq@163.com (Z.J.)

**Keywords:** infrared thermal imager, emissivity measurement, active irradiation, coating

## Abstract

To achieve rapid and precise non-contact measurements of coating emissivity at room temperature, a measurement method based on infrared thermal imager was proposed. By applying two irradiations with different energies to the target and reference surfaces, the influences of atmospheric transmittance, radiation of the target itself, environmental radiation, and atmospheric path radiation were eliminated, thereby enabling accurate emissivity measurement. Experiments were designed for validation with a mid-wave infrared thermal imager and a surface blackbody as the radiation source. Several combinations of irradiation energy were set to investigate the effects of average energy and energy difference between the two irradiations on the measured results. The normal emissivity of the coated sample plate in the mid-wave band was measured to generate the image of coating surface emissivity. Then, the emissivity measurement results of the proposed method were compared with those of the energy method and the point emissivity measuring instrument under the same conditions, and the comparison indicated that the proposed method can effectively measure the emissivity of coating. Some factors causing measurement errors were analyzed. Finally, an experiment was designed to compare the measurement speed between the proposed method and the currently used methods, and the experimental results were analyzed.

## 1. Introduction

Coatings are often applied on the surface of objects to alter the surface characteristics for protection, insulation, camouflage, or other purposes. With the passage of service time and owing to continuous influences of environmental factors, damages, such as aging, wear, and scratching, are prone to occur, which can cause adverse effects on coating performance [1]. Infrared emissivity is one of the important parameters for characterizing the coating performance, which generally changes if the coating is damaged. An accurate measurement of coating surface emissivity can locate the damaged sites on the coating and inform the degree of damage, which is essential in guiding the repair and replacement of the coating.

Infrared emissivity measurement methods are typically divided into two categories, namely, direct and indirect methods. Direct methods mainly include calorimetry [2], the energy method [3], and the multi-wavelength method [4], whereas indirect methods are mostly based on reflectometry [5]. A variety of emissivity measuring instruments have been designed according to these principles [6,7,8,9], which have achieved good measurement performance at high temperatures. However, these methods and instruments are insufficient when applied at room temperature, which has spurred the investigation of emissivity measurements specifically at room-temperature environments. Currently, the methods available for measuring coating emissivity at room temperature include integrating sphere reflectometry [10], the dual-band method [11], the variable-environment radiation method [12], and the energy method. Integrating sphere reflectometry is precise and low in environmental sensitivity, but it only allows contact measurements and has a limited measurement area. The dual-band method can realize non-contact, large-area measurements, but only with complex apparatuses. Moreover, it assumes that the emissivity of the object surface is equal in two adjacent bands, which is bound to reduce the measurement accuracy, and the emissivity of the whole band we need cannot be obtained by this method. The variable-environment radiation method can also realize non-contact measurements in a large scale, but it requires accurate knowledge of environmental radiation, which puts a stringent requirement on the equipment and environment. The energy method can achieve accurate measurement of emissivity at room temperature, but it must accurately measure the temperature of the target surface, usually by pasting a temperature sensor on the target, which is not allowed for some fragile or hard-to-contact surfaces. In summary, the existing methods are not well suited for precise non-contact emissivity measurements in room-temperature environments. In this study, combining the concept of reference body in the dual-band method and the basic thinking of the variable-environment radiation method, a method for measuring coating emissivity based on active irradiation was proposed. A mid-wave infrared thermal imager was used to measure the normal emissivity within 3–5 μm of a coated plate at room temperature, generating an infrared emissivity image of the sample and calculating the measurement deviation of the method with some existing methods.

Compared with the energy method and integrating sphere reflectometry, this method does not need to contact the target surface or paste the temperature sensor on the target, thus avoiding the damage to the target surface. Compared with the dual-wavelength method, this method only focuses on the measurement band, and there is no need to introduce the assumption that the emissivity of two adjacent bands is equal. Additionally, compared to the traditional reflection method and the variable-environment radiation method, this method does not need to calculate the radiation energy, but it is calculated by the difference of radiance reflected by two applied irradiances on the surface of the target and the reference body. At the same time, this method also eliminates the influence of atmospheric transmittance, radiation of the target itself, environmental radiation and atmospheric path radiation, and realizes a more accurate measurement. Moreover, the influence of the difference and average energy of applied irradiation energy on the measurement is also studied.

## 2. Methods

### 2.1. Principle of Measurement

When active irradiation is applied to a coating, the coating surface radiation received by the thermal imager contains the additional information about coating reflectivity. For an opaque object, a definite relationship exists between the reflectivity and emissivity. Therefore, two sets of relationships can be obtained by applying two active irradiations with different energies to the coating, which can yield the coating emissivity by eliminating the unknown parameters. The specific principle is as follows.

Set the temperature of the coating as T. When no active irradiation is applied, the radiance L(Δλ) of the coating surface in the band of Δλ that is received by the infrared thermal imager is expressed as [13]

(1) $$L\left(\Delta \lambda \right)=\tau \left(\Delta \lambda \right)\left(L_{O}\left(\Delta \lambda \right)+\rho \left(\Delta \lambda \right)\cdot L_{E}\left(\Delta \lambda \right)\right)+L_{A}\left(\Delta \lambda \right)$$

where τ(Δλ) is the mean atmospheric transmittance within Δλ; $L_{O}\left(\Delta \lambda \right)$ is the radiance within Δλ emitted by the coating itself; ρ(Δλ) is the reflectivity of the coating within Δλ; $L_{E}\left(\Delta \lambda \right)$ is the radiance of the environment within Δλ; $L_{A}\left(\Delta \lambda \right)$ is the atmospheric path radiance within Δλ.

$L_{O}\left(\Delta \lambda \right)$ in Equation (1) is related to the emissivity and temperature of the coating, which can be expressed as

(2) $$L_{O}\left(\Delta \lambda \right)=\varepsilon \left(\Delta \lambda \right)\cdot L_{b}\left(T,\Delta \lambda \right)$$

where ε(Δλ) is the emissivity of the coating within Δλ; $L_{b}\left(T,\Delta \lambda \right)$ is the radiance of a blackbody with a temperature of T within Δλ. For coatings with unknown emissivity and temperature, it is difficult to obtain a direct measurement of $L_{O}\left(\Delta \lambda \right)$.

First, active irradiation with a radiance of $L_{R1}\left(\Delta \lambda \right)$ is applied to the coating surface. Due to the short irradiation time, the coating surface temperature is deemed unchanged, and the coating surface radiance $L_{1}\left(\Delta \lambda \right)$ within Δλ received by the thermal imager is expressed as

(3) $$L_{1}\left(\Delta \lambda \right)=\tau \left(\Delta \lambda \right)\left[L_{O}\left(\Delta \lambda \right)+\rho \left(\Delta \lambda \right)\cdot \left(L_{E}\left(\Delta \lambda \right)+L_{R1}\left(\Delta \lambda \right)\right)\right]+L_{A}\left(\Delta \lambda \right)$$

Next, another active irradiation with a radiance of $L_{R2}\left(\Delta \lambda \right)$ is applied to the coating surface under the same environment. Similarly, the coating surface temperature is considered to be unchanged. The coating surface radiance $L_{2}\left(\Delta \lambda \right)$ within Δλ received by the thermal imager becomes

(4) $$L_{2}\left(\Delta \lambda \right)=\tau \left(\Delta \lambda \right)\left[L_{O}\left(\Delta \lambda \right)+\rho \left(\Delta \lambda \right)\cdot \left(L_{E}\left(\Delta \lambda \right)+L_{R2}\left(\Delta \lambda \right)\right)\right]+L_{A}\left(\Delta \lambda \right)$$

$L_{O}\left(\Delta \lambda \right)$, $L_{E}\left(\Delta \lambda \right)$, and $L_{A}\left(\Delta \lambda \right)$ in Equations (3) and (4) are difficult to measure directly, so these quantities are eliminated by subtracting Equations (3) and (4).

Equation (4) minus Equation (3)

(5) $$\rho \left(\Delta \lambda \right)=\frac{L_{2}\left(\Delta \lambda \right)-L_{1}\left(\Delta \lambda \right)}{\tau \left(\Delta \lambda \right)\cdot \left(L_{R2}\left(\Delta \lambda \right)-L_{R1}\left(\Delta \lambda \right)\right)}$$

For an opaque object, only energy absorption and reflection are involved when a radiant energy is incident on it. According to the law of energy conservation and Kirchhoff’s law, α+ρ=1, where α is the absorptivity, and ρ is the reflectivity. When the temperature of the object is constant, the amount of energy it absorbs equals the energy it emits, i.e., ε=α, so ε=1−ρ. Substituting this into Equation (5) obtains

(6) $$\varepsilon \left(\Delta \lambda \right)=1-\rho \left(\Delta \lambda \right)=1-\frac{L_{2}\left(\Delta \lambda \right)-L_{1}\left(\Delta \lambda \right)}{\tau \left(\Delta \lambda \right)\cdot \left(L_{R2}\left(\Delta \lambda \right)-L_{R1}\left(\Delta \lambda \right)\right)}$$

Equation (6) is the measurement formula for coating surface emissivity, which indicates that the emissivity ε(Δλ) of the coating surface within Δλ can be obtained as long as the atmospheric transmittance τ(Δλ), the radiances of the two active irradiations, $L_{R1}\left(\Delta \lambda \right)$ and $L_{R2}\left(\Delta \lambda \right)$, and the corresponding radiances received by the infrared thermal imager, $L_{1}\left(\Delta \lambda \right)$ and $L_{2}\left(\Delta \lambda \right)$, are known.

Since the measurement time is very short and the measurement distance is relatively close, the atmospheric distribution in the environment can be deemed uniform and fixed. Therefore, the atmospheric transmittance τ(Δλ), environment radiance $L_{E}\left(\Delta \lambda \right)$, and atmospheric path radiance $L_{A}\left(\Delta \lambda \right)$ remain unchanged during the whole measurement process. τ(Δλ) can be calculated by software, such as MODTRAN [14], or obtained by experimental calibration. However, the specific value of τ(Δλ) is not required in the proposed method, and the reason will be given below.

$L_{1}\left(\Delta \lambda \right)$ and $L_{2}\left(\Delta \lambda \right)$ in Equation (6) are directly measured, and τ(Δλ) is first taken as a known quantity. Then, the only undetermined quantity is the difference between $L_{R2}\left(\Delta \lambda \right)$ and $L_{R1}\left(\Delta \lambda \right)$, which can be determined by the following method.

According to Equation (5), the difference in radiance between the two active irradiances can be expressed as

(7) $$L_{R2}\left(\Delta \lambda \right)-L_{R1}\left(\Delta \lambda \right)=\frac{L_{2}\left(\Delta \lambda \right)-L_{1}\left(\Delta \lambda \right)}{\tau \left(\Delta \lambda \right)\cdot \rho \left(\Delta \lambda \right)}$$

Equation (7) indicates that the radiance difference $L_{R2}\left(\Delta \lambda \right)-L_{R1}\left(\Delta \lambda \right)$ can be calculated as long as the target reflectivity ρ(Δλ) within Δλ is known. Since the reflectivity of the coating to be measured is unknown, a reference body with a known reflectivity needs to be introduced.

Supposing the reflectivity of the reference body within Δλ to be $\rho_{C}\left(\Delta \lambda \right)$, place the reference body next to the coating plate to be measured and make the distance between the reference body and the imager equal to that between the coating plate and the imager. This step ensures that reference body and the sample are in the same environment, so Equations (1) and (2) can also be applied to the reference body. When active irradiation is applied to the coating surface, it is also irradiated on the reference body surface, so Equations (3) and (4) are also applicable to the reference body. Similarly, removing the unknown parameters by subtracting the two equations, the difference in radiance between the two active irradiations can be obtained as

(8) $$L_{R2}\left(\Delta \lambda \right)-L_{R1}\left(\Delta \lambda \right)=\frac{L_{C2}\left(\Delta \lambda \right)-L_{C1}\left(\Delta \lambda \right)}{\tau \left(\Delta \lambda \right)\cdot \rho_{C}\left(\Delta \lambda \right)}$$

where $L_{C1}\left(\Delta \lambda \right)$ and $L_{C2}\left(\Delta \lambda \right)$ are the radiances of the reference body surface received by the thermal imager under an active irradiation of $L_{R1}\left(\Delta \lambda \right)$ and $L_{R2}\left(\Delta \lambda \right)$, respectively. Finally, the coating surface emissivity ε(Δλ) is calculated by substituting Equation (8) into Equation (6):

(9) $$\varepsilon \left(\Delta \lambda \right)=1-\rho_{C}\left(\Delta \lambda \right)\cdot \frac{L_{2}\left(\Delta \lambda \right)-L_{1}\left(\Delta \lambda \right)}{L_{C2}\left(\Delta \lambda \right)-L_{C1}\left(\Delta \lambda \right)}$$

### 2.2. Type of Measured Emissivity

The object emissivity can be categorized into hemispherical emissivity and directional emissivity. The normal emissivity is the directional emissivity in the normal direction of the material surface. According to the emissivity in the wavelength range, it can be further subdivided into full emissivity, spectral emissivity, and band emissivity. Due to the existence of infrared atmospheric window, the infrared thermal imagers generally work in the bands of 1–2.5 μm (short wave), 3–5 μm (mid wave), or 8–14 μm (long wave), so the general research focuses on the emissivity of these bands.

According to the proposed method, measurements are recorded with a thermal imager, and Δλ is set as its response band. What the thermal imager receives is only the energy that the object radiates within a small solid angle, so the measured radiance is the amount in a certain direction. When the imager’s axis coincides with the normal of the object surface, and the measurement distance is far relative to the object size, the measurement direction can be considered normal, i.e., $L_{1}\left(\Delta \lambda \right)$, $L_{2}\left(\Delta \lambda \right)$, $L_{C1}\left(\Delta \lambda \right)$, and $L_{C2}\left(\Delta \lambda \right)$ in Equation (9) are normal radiances. Let

(10) $$K=\frac{L_{2}\left(\Delta \lambda \right)-L_{1}\left(\Delta \lambda \right)}{L_{C2}\left(\Delta \lambda \right)-L_{C1}\left(\Delta \lambda \right)},$$

so

(11) $$\varepsilon_{⊥}=1-K\cdot \rho_{C⊥}$$

where $\varepsilon_{⊥}$ is the measured normal emissivity, and $\rho_{C⊥}$ is the normal reflectivity of the reference body.

### 2.3. Theoretical Analysis

It is known from Equation (9) that the atmospheric transmittance τ(Δλ) is eliminated in the process of substitution, which is the reason why it is unnecessary to know the specific value of τ(Δλ), as mentioned in Section 2.1. Besides, although $\rho_{C}\left(\Delta \lambda \right)$ is a known quantity, the other 4 radiances in Equation (9) can be directly measured by the thermal imager. Therefore, the proposed method can measure the emissivity without knowing the real temperature of the target.

Compared with the traditional reflection method for measuring emissivity, this method does not need to calculate the single irradiation energy of the radiation source but only focuses on the difference between the two applied irradiation energies. Therefore, in application, only one or more radiation sources that can stably emit infrared radiation of corresponding wavelength need to be used. At the same time, the significant difference is that this method uses infrared thermal imager as the sensor, and after data processing, the emissivity image of the target can be obtained instead of a single value, which is convenient for application in the field of coating damage detection and repair.

The proposed method is close to the bi-background method [15] in terms of principle, and the final form of the emissivity calculation formulae is also similar. However, in the bi-background method, a blackbody is involved in altering the environment radiation and constructing different environmental backgrounds. The proposed method, on the other hand, measures by solving the difference when two active irradiations with different energies are applied in the same environment. Furthermore, the effect of atmospheric transmittance is canceled, since a reference body is used.

For example, in Reference [16], two backgrounds, the field environment background and the background after the introduction of the blackbody, were constructed, and the object emissivity was measured using a radiation thermometer. This approach was, in practice, a special case of the proposed method when $L_{R1}\left(\Delta \lambda \right)=0$. In other words, only one active irradiation was applied, and the calculation formula of ε(Δλ) can be constructed according to Equations (1) and (3). However, this method is unsuitable for thermal imager measurements at room temperature because the radiation power received by the thermal imager at room temperature is independent of the surface emissivity for opaque objects if environmental radiation is considered. This radiation power is also close to the background radiation, which decreases the signal-to-noise ratio of the infrared thermal imager and increases the measurement error, making it difficult to achieve precise measurements.

For another example, in Reference [12], two different blackbody radiations were directly used as background environment radiations to construct the bi-background, and a long-wave thermal imager was used to measure the emissivity. This method did not require the use of a reference body, since the calibrated value of blackbody radiance was taken as the environmental radiance, but it failed to eliminate the atmospheric transmittance from the formula. Instead, the method assumed a constant atmospheric transmittance of 1 that was only applicable to close-range tests, which created some measurement errors. Moreover, the calibrated blackbody radiance was directly calculated as the environment radiation, which would also cause some errors unless the measurement was performed in an infrared darkroom.

The proposed method applies two active irradiations with different energies to the surfaces of the coating and reference body to enhance the contrast of radiation before and after irradiation. In the meantime, the influences of atmospheric transmittance τ(Δλ), coating radiance $L_{O}\left(\Delta \lambda \right)$, environment radiance $L_{E}\left(\Delta \lambda \right)$, and atmospheric path radiance $L_{A}\left(\Delta \lambda \right)$ are eliminated, thus achieving accurate measurement of coating surface emissivity.

## 3. Experimental Equipment

### 3.1. Instruments and Setup

**Figure 1:**
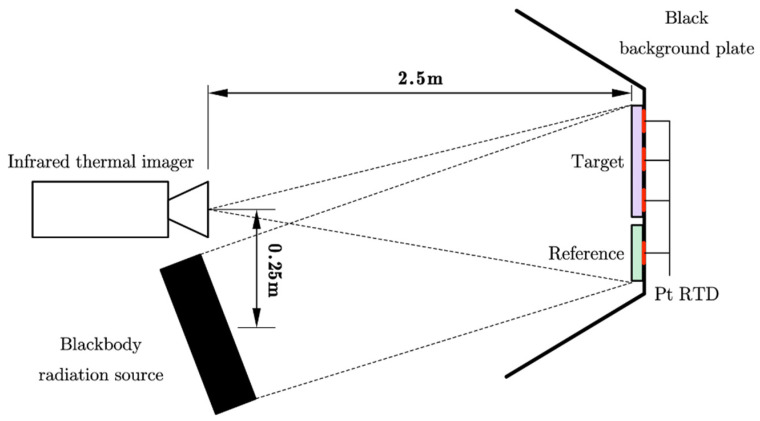
Schematic diagram of experimental setup.

A 640 × 512 pixels, 25 Hz cooled mid-wave infrared thermal imager (3–5 μm) was used to measure the target radiance. A 50 mm × 50 mm surface source blackbody S_1_ with an emissivity of 0.93 and a temperature control accuracy ≤10 mK was used as the benchmark for radiance calibration of the thermal imager. A 200 mm × 200 mm surface source blackbody S_2_ with an emissivity of 0.93 was used as the radiation source. The target was a coated metal plate, its matrix was TC4 titanium alloy, and its coating material was resin-matrix composite. A black hardboard with an average emissivity of 0.962 was used as the measurement background to avoid external disturbances. A uniform coated plate with an average normal emissivity of 0.589 in the range of 3–5 μm was used as the reference body, the surface of which had reflection characteristics similar to those of the target with intact coating. A patch platinum resistance temperature detector (Pt RTD) was pasted behind the target to be measured and the reference body to measure the temperature of the object, which was used to calculate the target emissivity by energy method. The experimental setup is shown in Figure 1.

The experiments were conducted in a room-temperature environment with no abrupt changes during the measurement. The target was placed close to and on the same plane as the reference body, and the plane’s normal was parallel to the imager’s optical axis. The target and the blackbody S_2_ were placed opposite to each other, with a suitable distance and angle relative to S_2_, so that their surfaces were both irradiated. The distance between the radiation plane center and the infrared thermal imager was 0.25 m, and the imager was 2.5 m away from the target and reference body plane. The target and reference body were ensured to be present simultaneously in the field of view of the imager and imaged clearly by adjusting the focal length.

### 3.2. Radiation Calibration of the Infrared Thermal Imager

The infrared thermal imager works by converting the infrared radiation energy into the electrical signal with an infrared photosensor, which is based on the theory of blackbody radiation. To establish the relationship between the output signal and the input radiation energy, the standard blackbody radiation source needs to be employed to calibrate the output response of the infrared thermal imager and then obtain its system response formula.

The calibration model for the infrared thermal imager is as follows [12,17]:

(12) G=A⋅L+B,

where G is the gray level output of the imager, which ranged from 0 to 2^16^-1 in this experiment; L is the radiance received by the imager; A and B are the response coefficients of the imager, which are related to the build of the imager, the structure of the circuit system, and the materials of light-sensitive components. So, these two values generally differ in different thermal imagers and change over time for the same imager. For cooled thermal imagers, A and B are relatively stable within a certain period. Therefore, the received radiance can be calculated from the output of the imager according to the following equation:

(13) $$L=\frac{G-B}{A}$$

The radiation calibrating process of the infrared thermal imager is as follows. Capture the surface source blackbody S_1_ with the infrared thermal imager and record the imager output and the corresponding blackbody temperature as the temperature was changed.

According to the Planck formula

(14) $$L_{b}=\frac{\varepsilon_{b}}{\pi }\int_{3}^{5}\frac{c_{1}}{\lambda^{5}}\cdot \frac{1}{e^{c_{2}/\lambda T}-1}\;d\lambda ,$$

where $\varepsilon_{b}$ is the emissivity of the surface source blackbody S_1_. Substituting the blackbody temperature yields the radiance within 3–5 µm at the corresponding temperature. The corresponding data of radiance and imager output are presented in Table 1.

**Figure 2:**
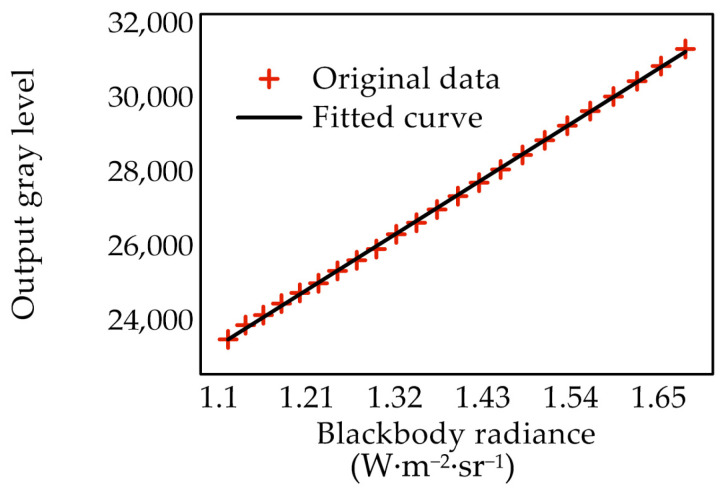
Calibration curve of thermal imager output and blackbody radiance.

The specific values of response coefficients A and B could be obtained by fitting, and the radiation calibration curve is shown in Figure 2.

The system response formula of the infrared thermal imager measured by the calibration experiment was

(15) G=13,531.0733L+8462.6663

The thermal imager worked continuously for 25 min during calibration, and the results demonstrated high linearity and stability in the imager response during measurement, meeting the measurement requirements.

## 4. Results and Discussion

### 4.1. Selection of Irradiation Combination

**Figure 3:**
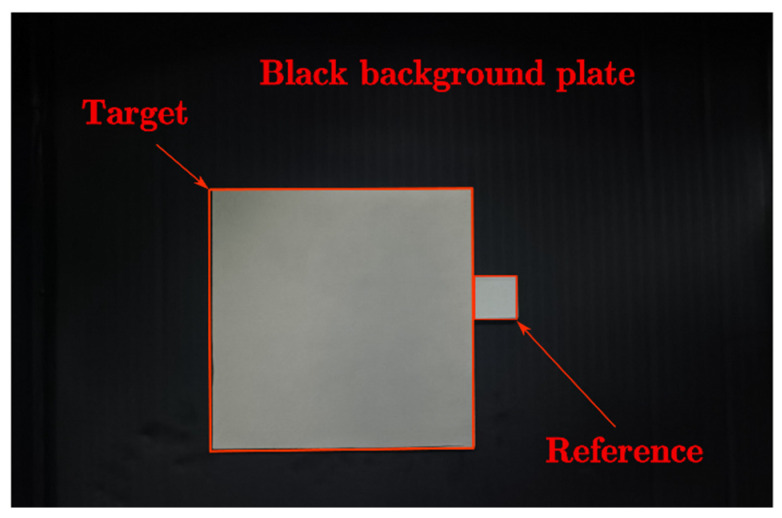
Image of the coating plate, reference body, and black background plate.

In this experiment, a metal plate with infrared low-emissivity coating was measured, which was closely attached to the reference body and pasted on the black background plate, as shown in Figure 3.

Five irradiation energies (denoted by the temperature of the radiation source) were selected, as displayed in Table 2.

**Figure 4:**
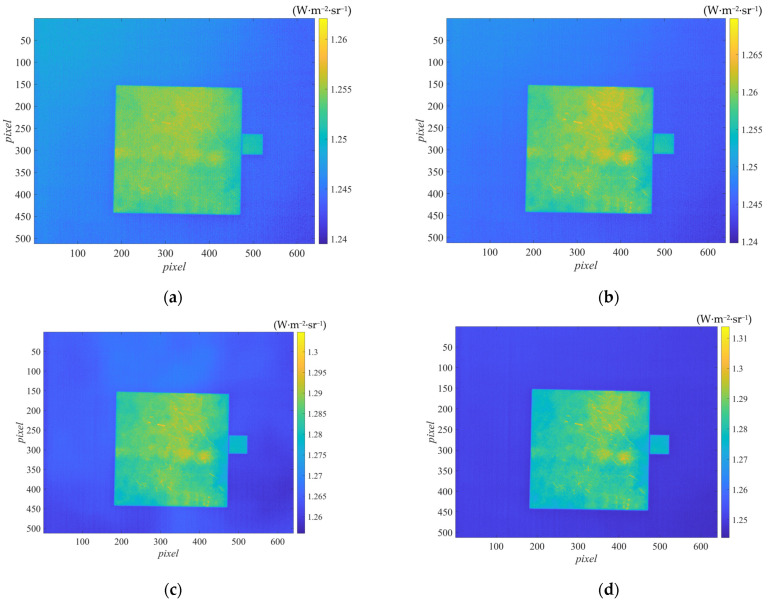

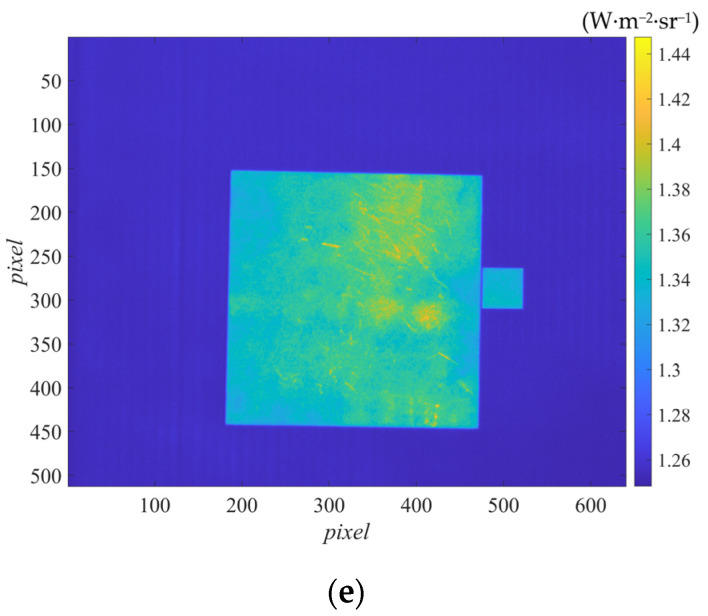
Radiance images output by the thermal imager at the five irradiation energies. (**a**–**e**) corresponded to the radiation source temperature of 50, 75, 100, 125, 150 °C, respectively.

The radiance (calibrated) images under the five irradiation energies were captured with the thermal imager and shown in Figure 4.

By comparing Figure 4a–e, it could be concluded that the higher the irradiation energy, the richer the image details, and the higher the contrast of different emissivity locations in the image. According to the measurement principle in Section 2.1, one set of emissivity could be measured for each combination of two irradiations. To explore which irradiation combination worked better, ten combinations were selected, as listed in Table 3.

**Figure 5:**
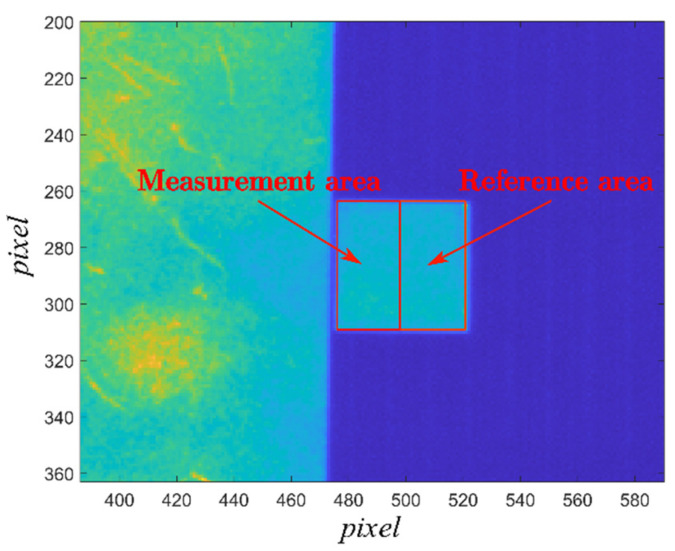
Diagram of selected reference area and measurement area on the reference body.

For convenient comparison, two small regions on the reference body were selected as the reference area and the measurement area, respectively, as shown in Figure 5.

Five measurements were recorded for each radiation combination. For each measurement, the average emissivity of the measurement area was calculated from the reference area selected on the reference body, and the following Formula (16) was used to calculate the standard deviation:

(16) $$S=\sqrt{\frac{\sum {\left(x_{i}-\bar{x}\right)}^{2}}{N-1}}$$

The results are shown in Table 4.

**Figure 6:**
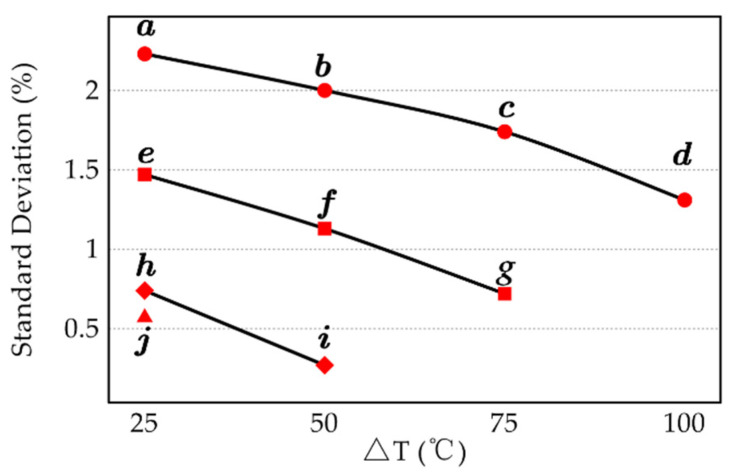
Relationship between temperature difference and standard deviation for the ten irradiation combinations.

**Figure 7:**
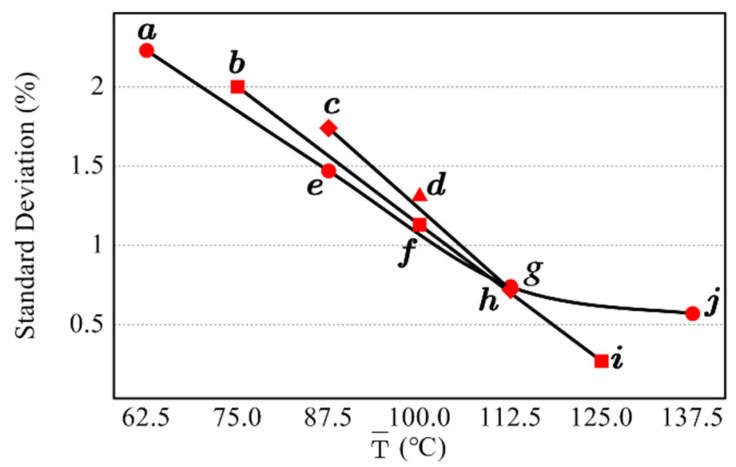
Relationship between average temperature and standard deviation for the ten irradiation combinations.

The results revealed that the proposed method was stable, with a standard deviation less than 2.5% in all irradiation combinations. Scatterplot of temperature difference and standard deviation was drawn, and points with the same lower temperature were connected, as presented in Figure 6. Scatterplot of average temperature and standard deviation was also drawn, and the points with an equal temperature difference between the two irradiations were connected, as presented in Figure 7.

Combining the information in Figure 6 and Figure 7, it could be concluded that increasing the average energy or the difference between the two irradiations could significantly improve the measurement result. This was because the radiation of the measured object was similar to that of the environment at room temperature, so the activity of the experimenter and the disturbances in the environment could significantly change the radiation of the target surface. Increasing the irradiation energy or the difference between the two irradiation energies could weaken the effects of personnel and environment disturbances and enhance the contrast between locations with different emissivity, thus improving the measurement results. For the five irradiation energies selected in the experiment, increasing the difference between the two irradiation energies would reduce one of them. According to comprehensive comparison, combination ***i*** was ultimately selected as the irradiation combination used to measure the coating plate.

### 4.2. Target Measurement

**Figure 8:**
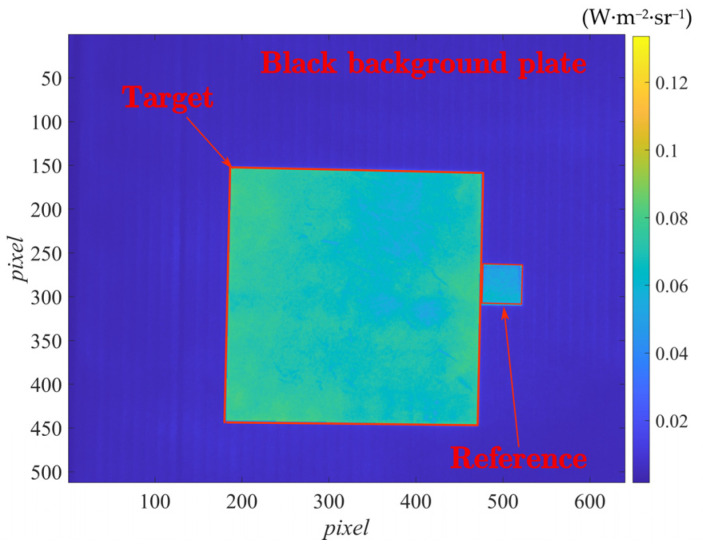
Radiance difference image obtained under irradiation combination ***i***.

Irradiation combination ***i*** involved irradiation ***5*** and irradiation ***3***. By subtracting the radiance image taken under irradiation ***3*** from that under irradiation ***5***, the difference image was obtained, and the reference body area was selected in the image, as shown in Figure 8.

**Figure 9:**
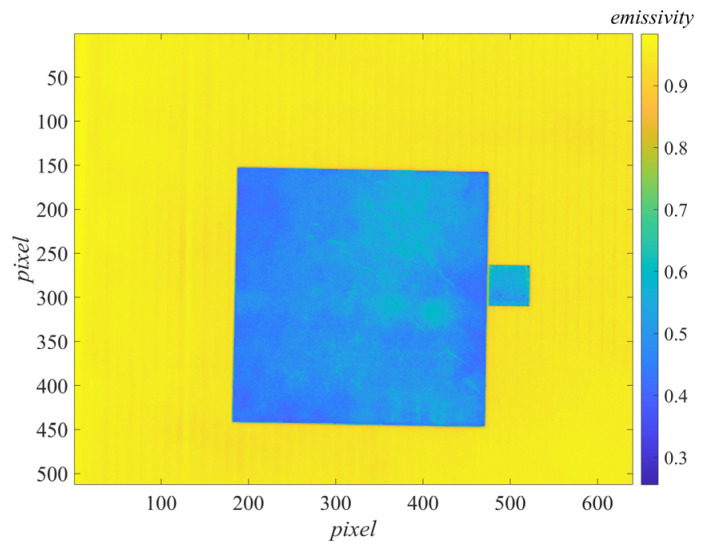
Measured emissivity image by our method.

By averaging the values of the reference body area in the difference image by pixels, $L_{C2}\left(\Delta \lambda \right)-L_{C1}\left(\Delta \lambda \right)$ in the emissivity measurement formula Equation (9) was obtained. The values of each pixel point in the difference image could be used as $L_{C2}\left(\Delta \lambda \right)-L_{C1}\left(\Delta \lambda \right)$, and the average reflectivity of the reference body $\rho_{C}=0.411$ was substituted. The emissivity image could be generated by pixel-to-pixel calculation according to Equation (9), as shown in Figure 9.

In order to test the effect of measurement, the results of this method are compared with those of the energy method. This article only briefly introduces the calculation steps of the energy method: shoot the target and reference body with the same thermal imager, ignoring the atmospheric influence. According to Equations (1) and (2), the radiance of the target can be expressed as

(17) $$L\left(\Delta \lambda \right)=\varepsilon \left(\Delta \lambda \right)\cdot L_{b}\left(T,\Delta \lambda \right)+\rho \left(\Delta \lambda \right)\cdot L_{E}\left(\Delta \lambda \right)$$

Similarly, the radiance of the reference body can be expressed as

(18) $$L_{C}\left(\Delta \lambda \right)=\varepsilon_{C}\left(\Delta \lambda \right)\cdot L_{b}\left(T_{C},\Delta \lambda \right)+\rho_{C}\left(\Delta \lambda \right)\cdot L_{E}\left(\Delta \lambda \right)$$

Through Equation (18), we can obtain

(19) $$L_{E}\left(\Delta \lambda \right)=\frac{L_{C}\left(\Delta \lambda \right)-\varepsilon_{C}\left(\Delta \lambda \right)\cdot L_{b}\left(T_{C},\Delta \lambda \right)}{\rho_{C}\left(\Delta \lambda \right)}$$

where $L_{C}\left(\Delta \lambda \right)$ is the measured value, $\varepsilon_{C}\left(\Delta \lambda \right)$ and $\rho_{C}\left(\Delta \lambda \right)$ are known, $T_{C}$ is obtained by the temperature sensor, and $L_{b}\left(T_{C},\Delta \lambda \right)$ is calculated by Equation (14).

Substituting Equation (19) into Equation (17) and according to the relationship ε=1−ρ described in Section 2.1, we can obtain:

(20) $$\varepsilon \left(\Delta \lambda \right)=\frac{\rho_{C}\left(\Delta \lambda \right)\cdot L\left(\Delta \lambda \right)-L_{C}\left(\Delta \lambda \right)+\varepsilon_{C}\left(\Delta \lambda \right)\cdot L_{b}\left(T_{C},\Delta \lambda \right)}{\rho_{C}\left(\Delta \lambda \right)\cdot L_{b}\left(T,\Delta \lambda \right)-L_{C}\left(\Delta \lambda \right)+\varepsilon_{C}\left(\Delta \lambda \right)\cdot L_{b}\left(T_{C},\Delta \lambda \right)}$$

**Figure 10:**
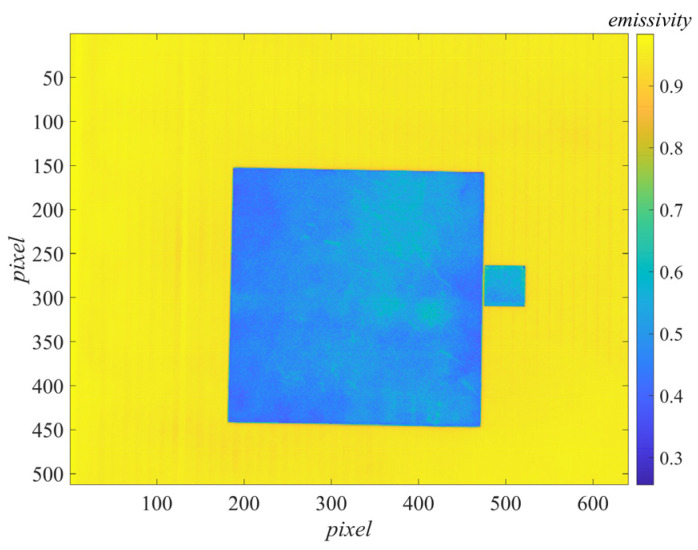
Measured emissivity image by the energy method.

Calculated pixel by pixel, the emissivity image obtained by the energy method can be generated according to Equation (20), as shown in Figure 10.

The damaged area (the greenish part of the target area in Figure 9 and Figure 10) could be clearly identified in the emissivity image, which contained scratches, wear, and inhomogeneities caused by insufficient coating spraying techniques. The reflection characteristics of these parts seriously deviate from the intact coating, resulting in a large deviation in emissivity measurement.

Let the emissivity represented by any pixel in the target area in Figure 9 be $\varepsilon_{a}$, and the emissivity represented by any pixel in the target area in Figure 10 be $\varepsilon_{b}$, and the relative deviation is defined as

(21) $$d=\frac{\left|\varepsilon_{a}-\varepsilon_{b}\right|}{\varepsilon_{b}}\times 100\%$$

**Figure 11:**
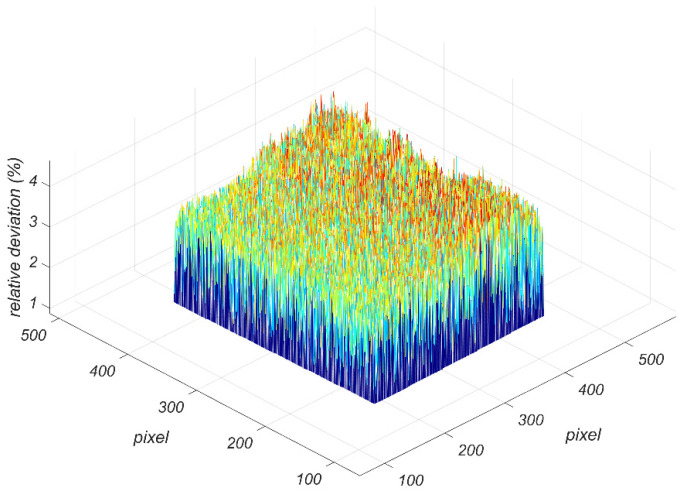
Three-dimensional relative deviation diagram.

Calculate all pixels in the target area to obtain a three-dimensional relative deviation diagram, as shown in Figure 11.

It can be seen from Figure 11 that the measurement results of this method are in good agreement with those of the energy method. In the target area, the maximum relative deviation of the measured emissivity is 4.49%, and the average relative deviation is 2.45%.

In addition, in order to further compare the measurement effect of this method, we also used the point emissivity measuring instrument based on the integrating sphere reflectometry [1,10] to measure the emissivity of some points in the target area. The specific comparison methods and results are as follows.

**Figure 12:**
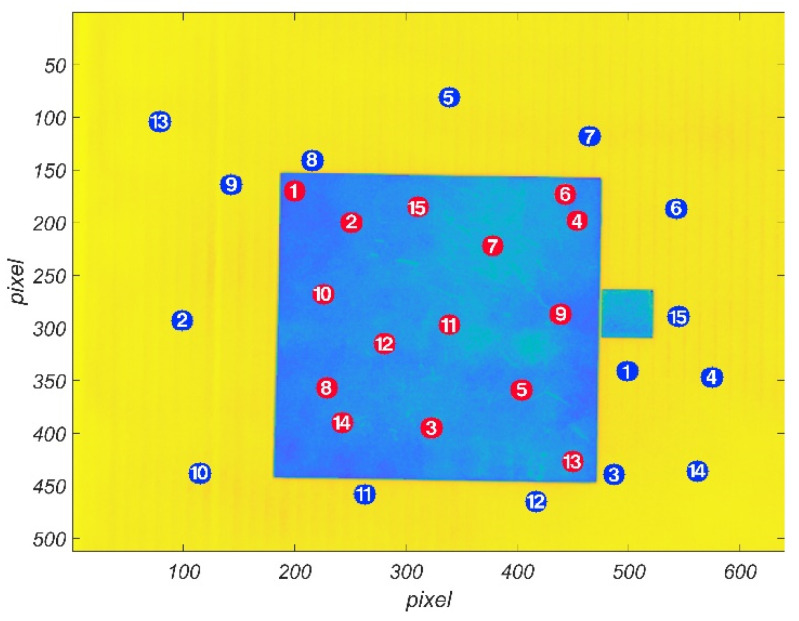
Randomly selected small circular measurement areas.

**Figure 13:**
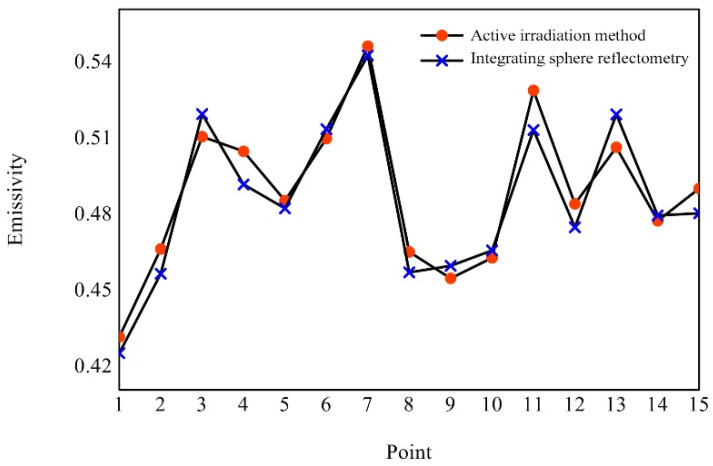
Comparison diagram of measurement results of target area.

Fifteen small circular measurement areas were randomly selected in the target area, as shown in Figure 12 (the red points). For each measurement area, the average emissivity of each pixel in the area was used as the measurement value of the central emissivity of the area. At the same time, the point emissivity measuring instrument was used to measure these points, and the measurement results are shown in Figure 13.

It can be seen from Figure 13 that the measurement results of this method are in good agreement with the measurement results of the point emissivity measuring instrument. The maximum relative deviation of the emissivity measurement values of the fifteen small circular measurement areas is 3.04%, and the average relative deviation is 1.57%.

**Figure 14:**
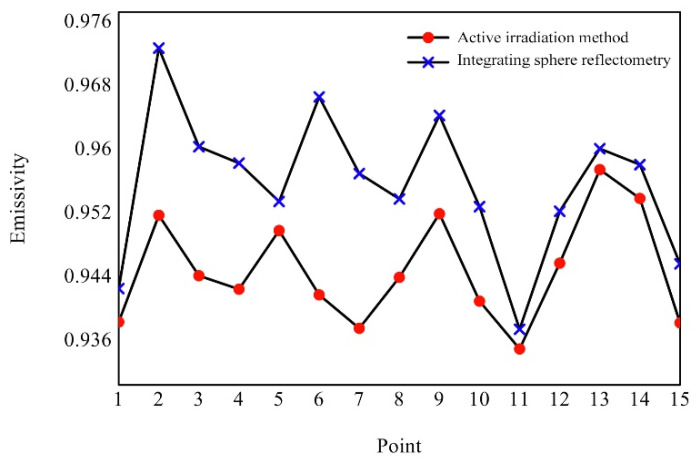
Comparison diagram of measurement results of black background plate area.

In addition to the target area, the black background plate area is also located in the field of view of the infrared thermal imager, and in the applied active irradiation, its emissivity can also be calculated by this method. Therefore, fifteen circular small measurement areas are selected on the black background plate area, as shown in Figure 12 (the blue points). At the same time, the emissivity of these points is measured by the point emissivity measuring instrument. The results are shown in Figure 14.

The maximum relative deviation of emissivity measurement at fifteen measurement positions selected in the black background plate area is 2.57%, and the average relative deviation is 1.13%. Although the relative deviation decreases, because the average emissivity of the black background plate is very high, at 0.962, the absolute deviation actually increases, and the measured value of this method in the selected area is generally lower than that of the point emissivity measuring instrument, mainly because the average emissivity of the black background plate is very high. For objects with a high emissivity, the absorptivity is also very high, according to Kirchhoff’s law, so the temperature rise of the object surface during measurement could not be ignored, which was inconsistent with the assumption in the measurement principle. Under this circumstance, $L_{2}\left(\Delta \lambda \right)$ at the higher irradiation energy and $L_{1}\left(\Delta \lambda \right)$ at the lower irradiation energy were both larger than the actual values, but the deviation of $L_{1}\left(\Delta \lambda \right)$ was smaller than that of $L_{2}\left(\Delta \lambda \right)$, so the resulting $L_{2}\left(\Delta \lambda \right)-L_{1}\left(\Delta \lambda \right)$ increased, thus making ε(Δλ) generally smaller. On the other hand, for objects with a high average emissivity, the average reflectivity is generally low. The average reflectivity of the black background plate was 0.038, so the radiance reflected by it at a low irradiation energy only occupied a small percentage of the overall radiance received by the thermal imager, which resulted in a low signal-to-noise ratio and thus a decreased measurement accuracy. Therefore, the reference body should have an emissivity as close to the target as possible. Furthermore, the measurement time should be as short as possible, and the average energy of the two irradiations should be reduced when measuring high-emissivity objects.

### 4.3. Comparison of Measurement Speed

The above show the effectiveness of the proposed method. In order to further illustrate the advantages of the proposed method, a simple comparative experiment was designed.

Five coated metal plates were used as measurement targets to simulate a large-area measurement target. The plates were placed separately, and each complete measurement required continuous measurement of each coated metal plate. The proposed method, energy method, and dual-band method were used to measure the targets. These three methods were all based on infrared thermal imager, so they could be compared. 

In the experiment, the same infrared thermal imager was used to measure the targets with these three methods, and the total time spend of each measurement and the time when each metal plate completed the measurement were recorded, as shown in Table 5. The preparation time at the beginning of each measurement was not recorded, and in each measurement, the time was recorded from the beginning of the measurement of the first metal plate to the completion of the measurement of the fifth metal plate. Moreover, the record only included the time of operation and data collection, excluding the time of data processing.

It can be seen from Table 5 that the proposed method is the fastest to complete, with a total time spend of 74.22 s and an average time of 14.84 s per plate. The energy method is the second, with a total time spend of 105.00 s and an average time of 21.00 s per plate. The dual-band method is the slowest, with a total time spend of 129.27 s and an average time of 25.85 s per plate. The analyses are as follows: 

The proposed method is simple to operate, and after completing the measurement preparation, the measurement can be completed only by applying two irradiations with different energy to the target. In this experiment, a uniform semitransparent attenuation sheet is used to change the irradiation energy, raise and fix the blackbody to a certain temperature, directly irradiate the target, collect the data under the first irradiation, and add an attenuation sheet in front of the blackbody. The measurement of a plate can be completed by collecting the data under the second irradiation, which saves the time of blackbody heating and cooling, and the irradiation energy switching can be completed within 1 s.

The energy method completes the measurement of the first board very quickly, but due to the need to paste multiple temperature sensors on the target, when measuring the second board, it is necessary to transfer the temperature sensors and wait for the sensor output to be stable, which greatly slows down the measurement time of continuous multiple targets. Increasing the number of temperature sensors or adopting faster temperature measurement methods may alleviate this problem; however, the cost and operation difficulty will be increased.

The dual-band method needs to go through the process of switching filters in the measurement of each plate, and our equipment needs more than 10 s to switch filters. Therefore, the dual-band method is generally slow in the measurement. The use of more advanced equipment may alleviate this problem, but the cost will also increase.

To sum up, the proposed method has great advantages over similar currently used methods in the speed and simplicity of continuous measurement of multiple targets. More importantly, the proposed method can realize real non-contact measurement, and the equipment used is simple. It has great application prospects in the emissivity measurement of some difficult-to-contact surfaces or easily damaged surfaces.

## 5. Conclusions

Targeting at rapid and precise non-contact measurements of coating emissivity at room temperature, a method based on active irradiation was proposed. The measuring principle of this method was described in detail, and the formula for measuring the emissivity was deduced. The experiment for measuring the coating emissivity based on active irradiation was designed and implemented. The experimental steps and data processing methods were described in detail. The effects of the average energy and energy difference between the two irradiations on the measurement results were investigated, obtaining an appropriate irradiation combination, which was used to measure the normal emissivity within a certain band. Additionally, an experiment was designed to compare the measurement speed of the proposed method with some similar currently used methods.

1. The proposed method did not require the measurement of target or reference body temperature. Besides, the effects of atmospheric transmittance τ(Δλ), coating radiance $L_{O}\left(\Delta \lambda \right)$, environment radiance $L_{E}\left(\Delta \lambda \right)$, and atmospheric path radiance $L_{A}\left(\Delta \lambda \right)$ that were difficult to measure, were considered. These quantities were eliminated by applying two active irradiations with different energies to the target and reference body under the same environment and subtracting the thermal imager outputs, thereby improving the accuracy of emissivity measurements;

2. The experimental results show that the measurement results of the proposed method are in good consistency with those of the energy method and the integrating sphere reflection method and can effectively measure the emissivity of the coating surface. It is also concluded that increasing the difference of two applied irradiation energies or increasing the average energy of two irradiations can improve the measurement effect;
3. The proposed method is simple to operate and can effectively realize rapid non-contact measurement. In the comparison of measurement speed, it can be concluded that the proposed method is significantly faster than similar currently used methods and has a good application prospect in the emissivity measurement of difficult-to-contact or easily damaged surfaces.

## Figures and Tables

**Table 1:** Blackbody temperature, radiance, and output gray level of the thermal imager.

Blackbody Temperature (°C)	Blackbody Radiance (W∙m^−2^∙sr^−1^)	Output Gray Level
15.000	1.1105	23,489.2
15.500	1.1324	23,873.5
16.000	1.1547	24,142.9
16.500	1.1773	24,453.9
17.000	1.2003	24,742.0
17.500	1.2237	25,000.1
18.000	1.2474	25,333.2
18.500	1.2715	25,618.3
19.000	1.2960	25,920.4
19.500	1.3209	26,317.2
20.000	1.3462	26,628.9
20.500	1.3719	26,984.5
21.000	1.3981	27,343.8
21.500	1.4246	27,707.1
22.000	1.4515	28,061.3
22.500	1.4789	28,451.4
23.000	1.5066	28,846.6
23.500	1.5349	29,239.2
24.000	1.5635	29,632.3
24.500	1.5926	30,024.7
25.000	1.6222	30,437.1
25.500	1.6522	30,844.2
26.000	1.6826	31,303.7

**Table 2:** Irradiation energy and type number.

Type Number	*1*	*2*	*3*	*4*	*5*
Radiation source temperature (°C)	50	75	100	125	150

**Table 3:** Irradiation combinations and type.

Type	*a*	*b*	*c*	*d*	*e*	*f*	*g*	*h*	*i*	*j*
Combination	2 and 1	3 and 1	4 and 1	5 and 1	3 and 2	4 and 2	5 and 2	4 and 3	5 and 3	5 and 4

**Table 4:** Emissivity and standard deviation of five measurements under different irradiation combinations.

Combination	1st	2nd	3rd	4th	5th	Average	Standard Deviation
***a***	0.6141	0.6314	0.5977	0.5748	0.6219	0.6080	2.23%
***b***	0.5678	0.5729	0.6145	0.6044	0.5937	0.5907	2.00%
***c***	0.6092	0.6076	0.5756	0.5727	0.5974	0.5925	1.74%
***d***	0.5956	0.6076	0.6152	0.5816	0.5928	0.5986	1.31%
***e***	0.6020	0.5955	0.5658	0.5869	0.5755	0.5851	1.47%
***f***	0.6154	0.5966	0.5933	0.5940	0.5849	0.5969	1.13%
***g***	0.6012	0.6112	0.5956	0.5922	0.5997	0.6000	0.72%
***h***	0.5954	0.5996	0.6125	0.6095	0.5987	0.6031	0.74%
***i***	0.5921	0.5947	0.5887	0.5891	0.5939	0.5917	0.27%
***j***	0.5853	0.5955	0.5975	0.6002	0.5927	0.5942	0.57%

**Table 5:** The completion time of each plate measurement, the total time spend of each measurement, and the average measurement time of each plate.

Method	1st	2nd	3rd	4th	5th	Total Time Spend	Average Time
Proposed method	11.16 s	27.16 s	42.25 s	58.31 s	74.22 s	74.22 s	14.84 s
Energy method	5.03 s	29.50 s	56.62 s	81.48 s	105.00 s	105.00 s	21.00 s
Dual-band method	19.56 s	46.17 s	69.89 s	93.86 s	129.27 s	129.27 s	25.85 s

## Data Availability

The data that support the findings of this study are available on request from the corresponding author on reasonable request.
